# On-bead purification and nanodisc reconstitution of human chemokine receptor complexes for structural and biophysical studies

**DOI:** 10.1016/j.xpro.2023.102460

**Published:** 2023-07-29

**Authors:** Siyi Gu, Mian Huang, Tracy M. Handel

**Affiliations:** 1 Skaggs School of Pharmacy and Pharmaceutical Sciences, University of California, San Diego, San Diego, CA 92093, USA

**Keywords:** Protein Biochemistry, Protein Expression and Purification

## Abstract

Chemokine receptors, a subfamily of G-protein-coupled receptors (GPCRs), are responsible for cell migration during physiological processes as well as in diseases like inflammation and cancers. Here, we present a protocol for solubilizing, purifying, and reconstituting complexes of chemokine receptors with their ligands in “nanodiscs,” soluble lipid bilayers that mimic the native environment of membrane receptors. The protocol yields chemokine receptor complexes with sufficient purity and yield for structural and biophysical studies and should be applicable to other GPCRs.

## Highlights

• Solubilization and purification of chemokine receptor complexes with chemokine ligands
• On-bead nanodisc reconstitution of chemokine receptor complexes
• Suitable for structural and biophysical studies of chemokine receptors
• Adaptable for the biochemical preparation of other G-protein-coupled receptors

## Abstract (editor highlights)

Publisher’s note: Undertaking any experimental protocol requires adherence to local institutional guidelines for laboratory safety and ethics.

## Graphical abstract

**Figure:**
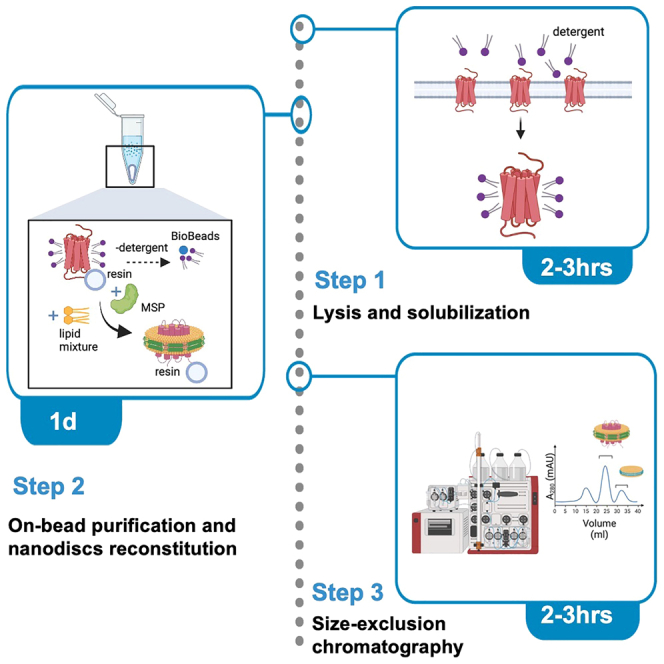

## Before you begin

Structural and biophysical studies of chemokine receptors in reconstituted systems can yield molecular information of how they function and lead to development of better therapeutics against these receptors.^1^ A prerequisite of such studies is that the biochemical preparations of the receptors are pure and stable. For example, for cryo-electron microscopy, sample purity generally needs to exceed 90% and the sample must withstand manipulations such as plunge freezing without loss of integrity. More recently, it has been shown that reconstituting GPCRs in bilayer lipid systems called nanodiscs can better stabilize the receptor of interest and reveal more information on receptor-lipid interactions, which may be critical for receptor function.^2^ Thus, a detailed protocol for efficient purification and nanodisc reconstitution of chemokine receptors should prove beneficial to researchers who want to obtain suitable samples for structural and biophysical studies. Compared to other widely used methods in which membrane proteins are first purified and then reconstituted into nanodiscs,^2^ this protocol combines the purification and reconstitution steps: the chemokine receptor is reconstituted into nanodiscs while bound to the affinity purification resin (the so called “on-bead” method). This method is especially beneficial to some chemokine receptors that are sensitive to detergent, since it shortens the time during which the receptor is in detergent micelles, which may mitigate detrimental effects of detergents^3^ and minimize protein loss and aggregation.

This protocol covers steps from the isolation of chemokine receptors from cellular membranes to reconstitution with chemokines or other ligands in nanodiscs. Before using this protocol, researchers need to design a DNA construct of the receptor of interest for expression and potentially modify the receptor for higher yield or improved stability by e.g., introducing stabilizing mutations or truncating flexible termini.^4^^,^^5^ Additionally, researchers can choose to either co-express the ligand of interest with the receptor, or add in purified ligands during the lysis step and/or subsequent purification steps. Since human chemokine receptors are seven-transmembrane proteins, expression systems such as insect or mammalian cells are recommended.^6^^,^^7^ Here, we discuss DNA construct design considerations and expression of chemokine receptors (alone or together with their ligands) using an insect cell expression system that is widely used and generally more economical than mammalian expression systems. We also cover preparation of components necessary for nanodisc reconstitution.

### Construct design considerations

**Timing: 2–5 days**

For expression of chemokine receptors in insect cells, the receptor and/or the chemokine (ligand) is cloned into the commonly used pFastbac vector with either the polH or GP64 promoter that drives expression. Important elements to include with the receptor sequence are as follows.

1. Signal sequence for proper insertion of the receptor into cell membranes: Commonly used sequences such as the hemagglutinin (HA) signal peptide (MKTIIALSYIFCLVFA) or the prolactin precursor signal sequence (MNIKGSPWKGSLLLLLVSNLLLCQSVAP) can be inserted at the N-terminus of the receptor and have been shown to effectively direct GPCRs including chemokine receptors to the insect cell surface.^8^^,^^9^^,^^10^ If the chemokine is co-expressed with the receptor, the endogenous or other chemokine signal sequence should also be included at the N-terminus of the chemokine for proper trafficking^11^; however, the signal sequence choice may determine if it is proteolytically processed in a manner that leaves the desired chemokine N-terminus.
2. Purification tags: Commonly used purification tags such as 10xHis, FLAG, StrepII or Rho 1D4 can be inserted at the N- or C-terminus (depending on tag) of the receptor sequence to enable affinity purification. Some tags are position sensitive: e.g., the Rho 1D4 tag needs to be inserted at the C-terminus of the receptor.^12^ This protocol is optimized using the commercially available Rho 1D4 purification tag, which enables very specific isolation of the tagged receptor.^12^ A protease cleavage site such as PreScission protease (PP) can be included between the receptor and the purification tag if the tag is undesired in the final product but is not necessary otherwise. No purification tag is recommended for the chemokine if co-expressed because the goal is to purify chemokine only when in complex with the receptor.
3. Modification of the receptor: Wild-type chemokine receptors are usually unstable after extraction from cell membranes. If necessary, stabilizing mutations can be incorporated into the receptor. Additionally, the flexible N or C-terminus of the receptor can be deleted. Both of these strategies have been shown to result in higher yield and better stability *in vitro* for some receptors.^4^^,^^13^ The mutated construct needs to be validated functionally (e.g., have comparable signaling and trafficking behaviors as the wild-type receptor) before use.

**Figure 1:**
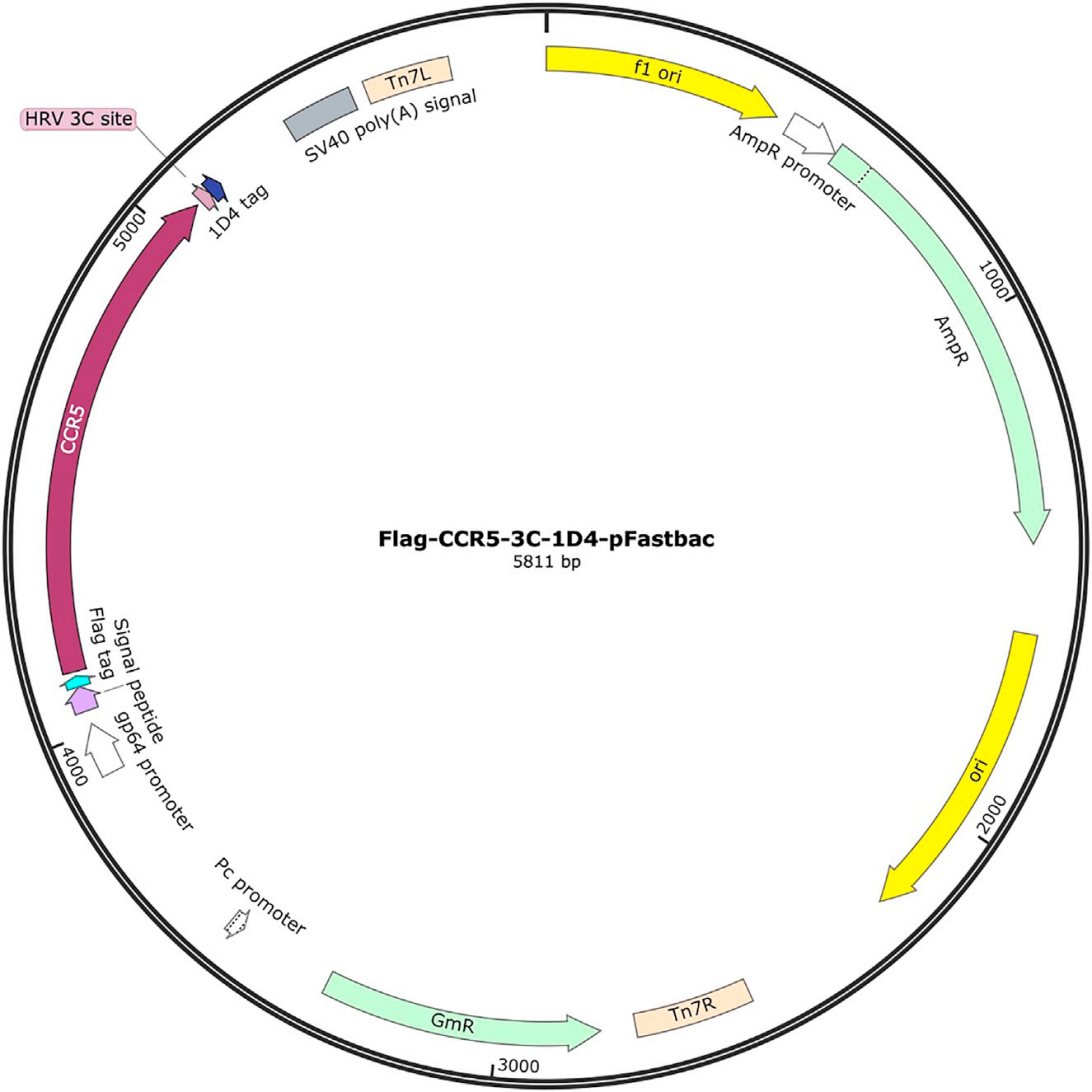
Insect cell expression plasmid for a chemokine receptor, CCR5 The pFastbac vector map shows CCR5 with an N-terminal HA signal peptide, an N-terminal Flag purification tag, and a C-terminal tag Rho 1D4 purification, under the control of a GP64 promotor. A 3C protease cleavage site between the receptor and 1D4 tag is also included for tag removal. The receptor sequence is truncated at the C-terminus to amino acid 320. The map was created using the SnapGene software.

An example of the pFastbac chemokine receptor expression vector is shown in Figure 1 and the sequence for the receptor construct is included in Data S1.

### Insect cell virus production and receptor expression

**Timing: 2 weeks**

We use the Invitrogen Bac-to-Bac system for co-expression of chemokine receptors with chemokines in insect cells. Recombinant baculovirus for the receptor (or the chemokine) is generated by transfecting recombinant bacmid DNA (e.g., the pFastbac vector containing the receptor/chemokine gene mentioned in the above step) into Sf9 cells using X-tremeGENE HP DNA Transfection Reagent (Roche) and Transfection Medium (Expression Systems). Cell suspensions are then incubated at 27°C for 4 days. The supernatant of the cell suspensions is collected as P0 viral stock and used to infect a new batch of sf9 cells for high titer baculovirus stock (P1) at 27°C. For expression, Sf9 cells at a cell density of 2–3 × 10^6^ cells/mL are infected with P1 virus at the desired multiplicity of infection (MOI) at 27°C. The MOI is typically 5–10 but needs to be optimized for each receptor/chemokine pair separately. Moreover, if co-expression of the receptor and the chemokine is desired, the ratio between the two is typically 1:1 but also needs to be optimized. At 48–72 h post infection, the cells are then harvested by centrifugation and the cell pellets are stored at −80°C until use. Researchers are advised to refer to the previously published protocol^14^ for extensive details.

### Preparation of nanodiscs components for receptor reconstitution

**Timing: 1–2 h**

For reconstitution of chemokine receptor complexes into nanodiscs, researchers first need to prepare a lipid mixture and membrane scaffold protein (MSP) stock, the two main components of the nanodisc system.^15^ For this protocol, we employ the widely used lipid mixture 60% 1-palmitoyl-2-oleoyl-*sn*-glycero-3-phosphocholine (POPC) : 40% 1-Palmitoyl-2-oleoyl-*sn*-glycero-3-(phospho-rac-(1-glycerol)) (POPG) w/w to mimic the plasma membrane lipid layer.^16^ Considering the size of chemokine receptors, we choose MSP1D1 which results in nanodiscs with an average diameter of 9–10 nm.^15^

4. POPC:POPG stocks are prepared at 63.7 mg/mL (83.8 mM total lipid mixture) in 180 mM sodium cholate as follows:
  a. Solubilize POPC and POPG in chloroform at 25 mg/mL as separate lipid stocks and store at −20°C until use.
  b. Depending on the total amount of lipid mixture stock being made, calculate the volume of POPC and POPG stock to mix. For example, in a total of 15 mg 60% POPC: 40% POPG mixture, 9 mg of POPC and 6 mg of POPG are needed, which corresponds to 360 μL 25 mg/mL POPC stock and 240 μL 25 mg/mL POPG stock.
  c. Add the required amount of the POPC and POPG stock into a glass test tube using a glass syringe,^16^ and dry the mixture using nitrogen gas until no visible liquid is present and a thin layer of glossy lipid appears on the bottom and sides of the glass tube.
  d. Further dry the lipid mixture by incubating the tube in a desiccator at 25°C for at least 1 h.
  e. Solubilize the dried lipid mixture with 180 mM sodium cholate solution at 63.7 mg/mL (83.8 mM), save at −80°C until use. Smaller aliquots can be prepared to avoid multiple freeze-thaw cycles.
5. We use commercially available MSP1D1 supplied in 5 mg lyophilized powder (see key resources table) and solubilize in 20 mM HEPES pH = 7.5, 150 mM NaCl buffer at 2 mg/mL (100 μM). Aliquots of 500 μL should be stored at −80°C until use.

**CRITICAL:** Chloroform can be toxic if inhaled or swallowed. Steps using chloroform should be performed with proper personal protective equipment using a fume hoods.

***Note:*** Some commercially available MSP1D1 proteins have purification tags (e.g., 6XHis tag). If the presence of the tag is undesirable, a product that does not have a purification tag can be chosen.

## Key resources table

**Table:** 

REAGENT or RESOURCE	SOURCE	IDENTIFIER
**Chemicals, peptides, and recombinant proteins**		

N-2-hydroxyethylpiperazine-N′-2-ethanesulfonic acid (HEPES, Fine White Crystals/Molecular Biology)	Fisher BioReagents^TM^	Cat#BP310
Sodium chloride (NaCl, Crystalline, Certified ACS)	Fisher Chemical^TM^	Cat#S271
Magnesium chloride Hexahydrate (MgCl_2_, Crystalline,Certified ACS)	Fisher Chemical^TM^	Cat#M33
Potassium chloride (KCl, White Crystals)	Fisher BioReagents^TM^	Cat#BP366
Iodoacetamide	Sigma-Aldrich	Cat#I1149
n-Dodecyl-β-D-maltopyranoside (DDM), Anagrade	Anatrace	Cat#D310
Cholesteryl hemisuccinate (CHS)	Sigma-Aldrich	Cat#C6512
Adenosine 5′-triphosphate disodium salt hydrate (ATP)	Sigma-Aldrich	Cat#6419
16:0-18:1 PC · 1-Palmitoyl-2-Oleoyl-sn-Glycero-3-Phosphocholine (POPC)	Anatrace	Cat#P516
P616: 1-Palmitoyl-2-Oleoyl-sn-Glycero-3-Phosphoglycerol, sodium salt (POPG)	Anatrace	Cat#P616
Chloroform	Sigma-Aldrich	Cat#288306
Rho 1D4 peptide	Cube Biotech	Cat#16201
3× Flag peptide (Lyophilized powder)	Sigma-Aldrich	Cat#F4799
MSP1D1	Cube Biotech	Cat#26112

**Critical commercial assays**		

cOmplete™, EDTA-free Protease Inhibitor Cocktail	Sigma-Aldrich	Cat#5056489001
PureCube Rho1D4 agarose	Cube Biotech	Cat#33102
Anti-Flag M2 Monoclonal Agarose Gel	Sigma-Aldrich	Cat#A2220
Bio-Beads SM-2 Adsorbents	Bio-Rad	Cat#1528920
Superdex 200 Increase 10/300 GL	Cytiva	Cat#28990944

**Software and algorithms**		

UNICORE 7	Cytiva	Cat#29128116

**Other**		

Centrifuge 5425, rotary knobs, refrigerated, with Rotor FA-24 × 2, 120 V/50–60 Hz (US)	Eppendorf	Cat#5406000445
Centrifuge 5810R, keypad, refrigerated, with Rotor A-4-62, 120 V/50–60 Hz (US), 20 A	Eppendorf	Cat#022627040
Avanti J-26XP High-performance centrifuge	Beckman Coulter	Cat#B22984
KIMBLE KONTES 40 mL Dounce All-Glass Tissue Grinders	DWK Life Sciences	Cat#885300-0040
Protein LoBind tube 0.5 mL/1.5 mL/2 mL	Eppendorf	Cat#0030108-434/442/450
Conical tube 15 mL	Biopioneer	Cat#CNT15
Conical tube 50 mL	VWR International	Cat#89039-656
Sartorius Vivaspin 6 mL Centrifugal Concentrators, MWCO: 100,000 d	Fisher Scientific	Cat#14558472
Akta Pure 25L	Cytiva	Cat#29018225

## Materials and equipment

**Table:** Low salt buffer (1 L)

Reagent	Final concentration	Amount
HEPES, pH = 7.2 (1 M)	10 mM	10 mL
MgCl_2_ (1 M)	10 mM	10 mL
KCl (2M)	20 mM	10 mL

Storage conditions: Store at RT until use

**Table:** 2× Solubilization buffer (50 mL)

Reagent	Final concentration	Amount
HEPES, PH = 7.2 (1 M)	100 mM	5 mL
NaCl (5 M)	300 mM	3 mL
DDM:CHS (10%)	2%	10 mL

Storage conditions: Freshly prepare before use; can store at 4°C for a week.

**Table:** Regular buffer (100 mL)

Reagent	Final concentration	Amount
HEPES, PH = 7.2 (1 M)	20 mM	2 mL
NaCl (5 M)	150 mM	3 mL
DDM:CHS (10%)	0.01%	0.1 mL

Storage conditions: Freshly prepare before use; can store at 4°C for a week.

• **HBS buffer:** 20 mM HEPES, PH=7.2; 150 mM NaCl

Store at RT.

• **10% DDM:CHS (5:1)** 50 mL: Dissolve 5 g DDM in 1 M Tris pH=8. Add 1 g CHS to the DDM solution, sonicate continuously at high power until the solution is translucent and then stir the solution at 25 °C until it is transparent.

Aliquot 10 mL in 15 mL conical tubes, store at −20°C until use.

The DDM and CHS powder should be equilibrated to 25°C before weighing.

## Step-by-step method details

### Cell lysis and receptor solubilization

**Timing: 2–3 h**

After harvesting chemokine receptor-expressing insect cells, the cell pellets can be frozen at −80°C until use. For purification, the cell pellets are either used directly or thawed (if frozen) and the chemokine receptors are extracted from the cell membranes using detergent. The protocol described below is optimized for 1 L of insect cells but can be scaled to accommodate other volumes.

1. Resuspend and thaw cell pellets with cold 40 mL Low Salt buffer on ice.
2. Dissolve:
  a. 1 tablet of protease inhibitor in 1 mL Low Salt buffer.
  b. 100 mg iodoacetamide in 1 mL Low Salt buffer
  c. If the chemokine is not co-expressed with the receptor, solubilize the ligand to the desired concentration in Low Salt buffer.

Add the above reagents to the cell solution, homogenize with a 40 mL glass dounce homogenizer 20–40 times, transfer to a 50 mL conical tube and rotate at 4°C for 20 min.

***Note:*** In the absence of co-expressed chemokine, chemokine receptors do not extract efficiently from cell membranes and are usually unstable after extraction. In this case, we recommend using high affinity small molecule ligands or purified chemokine at a concentration at least 1000-fold over the receptor:ligand dissociation constant (K_d_) for extraction.

3. Add 40 mL cold 2× Solubilization buffer containing 2 mM ATP to the cell lysate.
4. Stir the solubilization mixture in a glass beaker with a stir bar at 4°C for 3 h.
5. Centrifuge the solubilization mixture at 50,000–100,000 × *g*, 4°C for 30 min, and keep the supernatant on ice for subsequent purification and reconstitution steps.

***Note:*** The solubilized receptor is generally not stable enough for long-term storage at 4°C, and freeze-thawing can compromise the sample. Proceed immediately to the purification and reconstitution steps.

### On-bead purification and reconstitution

**Timing: 1 day**

Combining purification and nanodisc reconstitution steps while the receptor of interest is still bound to the affinity resin is critical for maintaining protein stability and avoiding protein loss during the process. We highly recommend using high specificity affinity purification systems such as Rhodopsin 1D4 or Flag tag. Here we describe our Rho 1D4 affinity purification strategy, which has been shown to be successful for many GPCRs including chemokine receptors.^17^^,^^18^ However, other affinity purification strategies such as Flag can be used with slight modifications of the protocol.

***Note:*** All steps should be performed on ice.

6. Wash the Rho 1D4 antibody resin by equilibrating 0.5 mL of the resin slurry (containing 50% resin) with 5 mL Regular buffer in each of two 50 mL conical tubes. Centrifuge the tubes at 300 × *g*, 4°C for 5 min; discard the supernatant.
7. Add the solubilized receptor from step 5 to the washed 1D4 resin. Rotate at 4°C for 2 h.
8. Centrifuge the receptor-1D4-resin mixture at 300 × *g*, 4°C for 5 min; then discard the supernatant.
9. Wash the resin in each tube with 20 mL Regular buffer; centrifuge for 5 min at 300 × *g*, 4°C and discard the supernatant.
10. Transfer the receptor-1D4-resin to two separate 2 mL Protein lo-bind tubes, wash one more time with 1 mL Regular buffer, centrifuge as above and discard the supernatant. The resin volume in each tube should be around 250 μL.
11. Dilute the POPC:POPG lipid stock to 2 mM (total lipid) with 1 mL Regular buffer, add 500 μL of the 2 mM lipid mixture to each centrifuge tube of receptor-1D4-resin, and rotate at 4°C for 30 min.
12. Centrifuge the lipid-receptor-1D4 resin mixture for 1 min at 300 × *g* and 4°C; discard the supernatant.
13. Add 500 μL 2 mg/mL MSP1D1 to each tube; rotate at 4°C for 30 min.
14. To remove detergent, add ∼20 mg of washed Bio-beads SM-2 resin to the MSP1D1-lipid-receptor-1D4 resin mixture; rotate at 4°C for 12–16 h.

***Note:*** The Bio-beads need to be reconstituted according to instructions from the manufacturer before use. The beads are first washed with methanol and then thoroughly washed with water. The beads can be stored in water before use.

15. Centrifuge the Bio-bead-MSP1D1-lipid-receptor-1D4 resin mixture for 1 min at 300 × *g*, 4°C; discard the supernatant.
16. Wash each tube of Bio-bead-MSP1D1-lipid-receptor-1D4 resin with 500 μL HBS buffer; centrifuge for 1 min at 300 × *g*, 4°C, and discard the supernatant.
17. Repeat step 16 twice.
18. To elute the nanodisc-solubilized receptor from the 1D4 resin, add 500 μL of 200 μM 1D4 peptide solubilized in HBS buffer to each tube and rotate at 4°C for 1 h.
19. To separate the nanodisc-solubilized receptor from the beads and the resin, centrifuge for 1 min at 300 × *g*, 4°C, and collect the supernatant from each 2 mL centrifuge tube in a 15 mL conical tube.
20. Repeat step 18–19 twice and combine all eluants in a single 15 mL conical tube for a total of approximately 3 mL of nanodisc-solubilized receptor. Store at 4°C for future use. The length of time the sample can be stored without loss of integrity is dependent on the receptor systems but in general it can be stored for at least a few days to a week.

***Alternatives:*** Other affinity purification strategies may be employed. For example, Flag-tagged receptor may be purified with M2 Flag antibody resin and eluted with 3XFlag peptide.

***Optional:*** Depending on the intended use of the reconstituted receptor, researchers can concentrate the elution collection to a desirable concentration and use directly. However, further purification using methods such as size exclusion chromatography (SEC) are strongly recommended.

***Note:*** Freezing of the reconstituted sample is not recommended.

### Size exclusion chromatography

**Timing: 2–3 h**

Nanodisc-solubilized chemokine receptors purified by affinity purification strategies, such as that described above, usually do not have sufficient purity for structural studies. A subsequent purification step using size exclusion chromatography (SEC) can substantially improve purity, and the SEC chromatograms provide a metric of the quality of the preparation. This protocol uses a Superdex S200 Increase 10/300 GL column configured for an AKTA^TM^ Fast Protein Liquid Chromatography (FPLC) system.

21. Concentrate the nanodisc-solubilized receptor from step 20 down to 500 μL using a 100 KDa membrane cutoff spin concentrator.
22. Centrifuge the sample for 15 min at 15,000–17,000 x *g* 4°C to clear particulates before applying to the FPLC system.
23. Equilibrate the ATKA FPLC system and the Superdex S200 column with HBS buffer at 4°C.

**CRITICAL:** Detergent in the FPLC system and the SEC column should be avoided by ensuring their removal with Biobeads (step 14 above).

24. Load the cleared and concentrated nanodisc-solubilized receptor sample onto the Superdex S200 column at a flow rate of 0.3–0.5 mL/min, 4°C.
25. Continue to pass HBS buffer through the column at a flow rate of 0.3–0.5 mL/min using 1.5 times the volume of the column bed. Collect 0.5-mL fractions and monitor the absorbance at 280 nm (A280) as an indicator of protein-containing fractions.
26. Collect SEC fractions based on the A280 readings, analyze the fractions using SDS-PAGE, and pool the desired fractions to yield the final product.

## Expected outcomes

**Figure 2:**
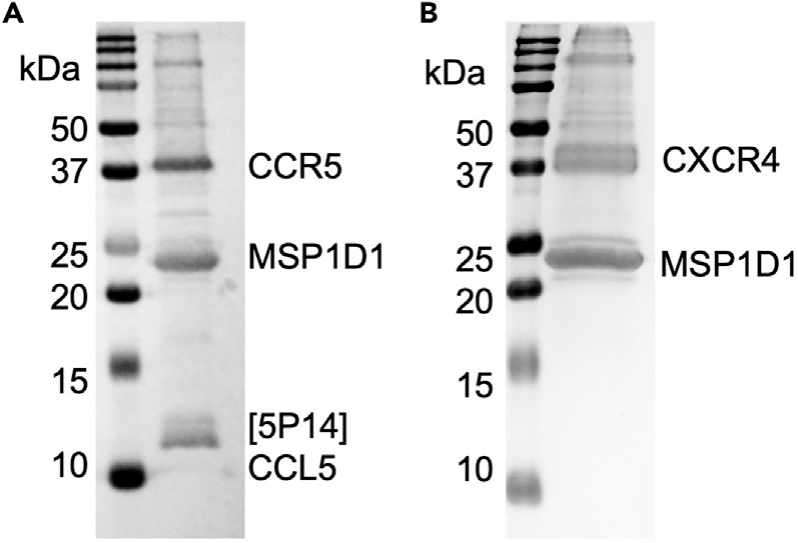
SDS-PAGE gels of purified and nanodisc-reconstituted CCR5-[5P14]CCL5 and CXCR4-IT1t (A) SDS-PAGE gel of CCR5-[5P14]CCL5 in nanodiscs eluted from Rho 1D4 antibody resin. The receptor was truncated at the C-terminus to amino acid 320, and was co-expressed with [5P14]CCL5 in insect cells. (B) SDS-PAGE gel of CXCR4-IT1t in nanodiscs eluted from M2 Flag antibody resin. The receptor was truncated at the C-terminus to amino acid 325 and was solubilized with 100 μM of the antagonist IT1t.

**Figure 3:**
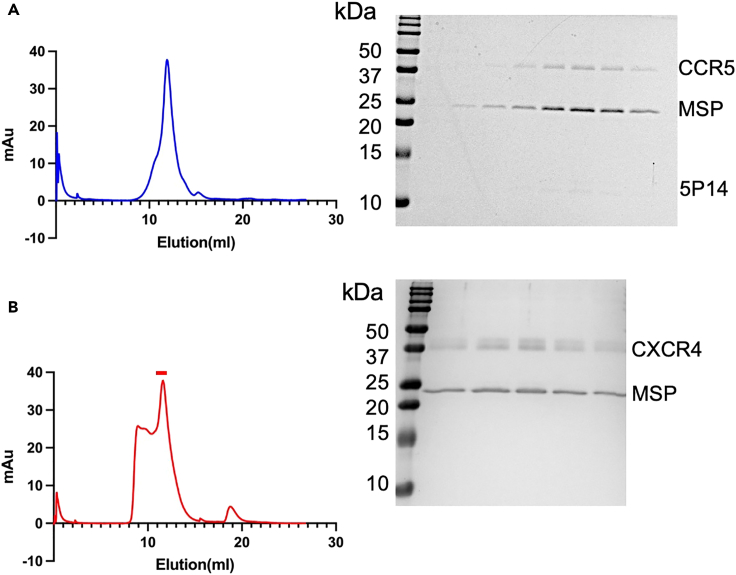
Size-Exclusion Chromatography of purified and nanodisc-reconstituted CCR5-[5P14]CCL5 and CXCR4-IT1t (A) SEC profile of CCR5-[5P14]CCL5 in nanodiscs (left) and SDS-PAGE gel of corresponding fractions from elution volumes 10–13.5 mL, 0.5 mL per fraction (right). (B) SEC profile of CXCR4-IT1t in nanodiscs (left) and SDS-PAGE gel of corresponding peak fractions (indicated by red bar) from elution volumes 11.5–13.5 mL, 0.5 mL per fraction (right).

The yield of nanodisc-reconstituted receptor is dependent on different receptor systems, but ideally should be over 50 μg of total protein per liter of expression and the purity should exceed 90%, which is suitable for most structural and biophysical studies. The SDS-PAGE should reveal prominent bands for the chemokine receptor at the expected molecular weight and MSP1D1 at approximately 20 kDa. If the chemokine is co-expressed with the receptor and has sufficient affinity to remain bound to the receptor during the purification and reconstitution steps, a band at the appropriate molecular weight for the specific chemokine is expected. Figure 2 shows SDS-PAGE gels of purified CCR5, which was co-expressed with the ligand [5P14]CCL5, and CXCR4 which was purified with the small molecule antagonist IT1t, both reconstituted in MSP1D1. The CCR5-[5P14]CCL5 complex was purified using 1D4 affinity chromatography while the CXCR4-IT1t complex was purified using Flag affinity chromatography. Size exclusion chromatography using a Superdex S200 column for the final purification step should reveal a single homogeneous peak at an elution volume corresponding to a 200–250 KDa protein. Peaks in the void volume or shoulders to the left of the main peak are indicative of aggregation and suggest the need for further optimization. Peaks at later elution volumes correspond to either empty nanodiscs or free MSP1D1 protein and should be avoided in the final product. SEC chromatograms of the CCR5 and CXCR4 complexes are shown in Figure 3.

## Limitations

Significant protein loss is inevitable during solubilization, purification, and especially nanodisc reconstitution steps. Thus, sufficient yields of nanodisc-reconstituted chemokine receptors rely on good expression in the first place. The on-bead purification and reconstitution method might not be very effective when using an affinity purification strategy with low specificity, such as Histidine tag purification. In addition, since the reconstitution step is done while the receptor is still bound to the affinity resin, there is no reliable way to quantify the receptor. Thus, precisely controlling the ratio of lipid and MSP to receptor during reconstitution can be challenging.

## Troubleshooting

### Problem 1

The yield of nanodisc-reconstituted receptor is low.

### Potential solution

• Increase the volume of insect cell membranes used for the preparation. For example, use 2–6 L of insect cells expressing the receptor of interest instead of 1 L.
• Improve the expression level of the receptor. Potential strategies involve engineering the receptor to make it more stable or less toxic to the expressing cells (i.e., truncation of flexible termini, introduction of stabilizing mutations, fusion with accessory protein domains).
• In step 2, use high affinity ligands for extraction. As mentioned previously, receptors are generally unstable after extraction from their membranes in the absence of ligands. Use of high affinity engineered chemokines or small molecules can improve extraction and stabilization of the receptor during purification and reconstitution and thereby mitigate protein loss. Chemokine antagonists are generally more stabilizing than agonists, especially in the absence of G proteins which generally stabilize agonist complexes. In addition to adding the ligand in the lysis step, researchers can also include the ligand in subsequent purification and reconstitution steps by adding it to the Regular buffer, Elution buffer and SEC running buffer.

### Problem 2

The efficiency of receptor solubilization is low.

### Potential solution

• In step 3, optimize the detergent used for solubilization. It has been shown that DDM:CHS or LMNG:CHS can be used for efficient solubilization and stabilization of GPCRs.^2^^,^^19^ However, it may be wise to explore other detergent options and additives if these detergent mixtures do not work well.
• In step 3, cholesterol may be added to the detergent mix to further stabilize the chemokine receptors instead of CHS. The concentration of cholesterol needs to be optimized.

### Problem 3

The purity of nanodisc-reconstituted receptor is low following affinity chromatography.

### Potential solution

• Histidine tag and other low specificity affinity chromatographic methods can result in the co-elution of significant amounts of protein contaminants. In steps 6 to 20 we recommend the use of high specificity affinity chromatographic methods.
• High salt concentrations (>500 mM NaCl) in the solubilization and purification buffers can negatively affect the purity of the reconstituted receptor. We recommend using 50–150 mM NaCl for these buffers.
• Before step 21, additional purification steps can be added following the on-bead purification and reconstitution step. More than one tag can be engineered in the receptor construct to enable a second round of affinity purification before or after the SEC step. However, this approach will inevitably decrease overall yield.

### Problem 4

The receptor is not properly reconstituted in nanodiscs or exhibits aggregation.

### Potential solution

• The amount of lipid mixture and MSP used in this protocol is optimized for saturating concentrations for most membrane proteins. For some receptors that express exceptionally well, researchers may need to increase the amount of lipid and MSP added in the reconstitution step (steps 11–14).
• For some receptors that express extremely well, in addition to increasing lipid and MSP, researchers can also increase the volume of buffers, affinity purification resin and Bio-beads to accommodate more receptor (steps 6–20). Alternatively, the amount of insect cell biomass can be reduced (for example use 0.5 L instead of 1 L).
• The type of MSP and lipids can be optimized for specific chemokine receptors. For example, natural phospholipid extracts or MSPs that are capable of forming larger size nanodiscs, such as MSP1E3D1, can be used.

### Problem 5

There is an excess of empty nanodiscs after affinity chromatography.

### Potential solution

• Increase wash volume or times (step 16–17) before receptor elution.
• Use SEC to separate empty and receptor-loaded nanodiscs.

## Resource availability

### Lead contact

Further information and requests for resources and reagents should be directed to and will be fulfilled by the lead contact, Tracy Handel (thandel@health.ucsd.edu).

### Materials availability

All materials generated in this study are available from the lead contact without restriction.

## Data Availability

• This study did not generate datasets. • This paper does not report original code. • Any additional information required to reanalyze the data reported in this paper is available from the lead contact upon request*.*
