# Supplementary material for: On-bead purification and nanodisc reconstitution of human chemokine receptor complexes for structural and biophysical studies
Source: STAR Protoc. 2023 Jul 29;4(3):102460. doi: 10.1016/j.xpro.2023.102460 (PMC10407235; doi:10.1016/j.xpro.2023.102460)
Supplement: Document S1. Data S1 [file mmc1.pdf]

Start (0) BsaAI BsaHI SpeI

5′ ttgtgtcacgtaggccagataacggtcgggtatataagacgcctcaacgctactagtaaatcagt 65
3′ aacacagtgcatccggtctattgccagcccatatattctgcggagttgcgatgatcatttagtca

gp64 promoter

StyI StuI* MflI* BstYI BamHI

cacaccaaggcttcaataaggaacacacaagcacgggcttctcctcaaaaggcctggatccatga 130
gtgtggttccgaagttattccttgtgtgttcgtgcccgaagaggagttttccggacctaggtact

gp64 promoter

1
M
Signal peptide

BbsI Bpu10I

agacgatcatcgccctgagctacatcttctgcctggtgttcgccgactacaaggacgatgatgac 195
tctgctagtagcgggactcgatgtagaagacggaccacaagcggctgatgttcctgctactactg

5 10 15 1 5
K T I I A L S Y I F C L V F A D Y K D D D D
Signal peptide Flag tag

AscI
BssHII

ggcgcgccgatggattatcaagtgtcaagtccaatctatgatattaattattatacatctgagcc 260
ccgcgcggctacctaatagttcacagttcaggttagatactataattaataatatgtagactcgg

1 5 10 15
G A P M D Y Q V S S P I Y D I N Y Y T S E P
CCR5

BsrGI

ctgccagaaaatcaatgtgaaacagatcgctgccaggctcctccctcccctgtacagcctggtgt 325
gacggtcttttagttacactttgtctagcgacggtccgaggagggaggggacatgtcggaccaca

20 25 30 35 40
C Q K I N V K Q I A A R L L P P L Y S L V
CCR5

BssSI
BssSαI EarI

ttattttcgggttcgtcggcaacatgctcgtgatcctcatcctcatcaattgcaagcggctgaag 390
aataaaagcccaagcagccgttgtacgagcactaggagtaggagtagttaacgttcgccgacttc

45 50 55 60
F I F G F V G N M L V I L I L I N C K R L K
CCR5

MscI*

agtatgacagacatctacctgctgaacctggccatctctgatctgttctttctgctcactgtgcc 455
tcatactgtctgtagatggacgacttggaccggtagagactagacaagaaagacgagtgacacgg

65 70 75 80
S M T D I Y L L N L A I S D L F F L L T V P
CCR5

BmrI

attctgggcacattacgcagcagcccagtgggacttcggaaataccatgtgtcagctcctgactg 520
taagacccgtgtaatgcgtcgtcgggtcaccctgaagcctttatggtacacagtcgaggactgac

85 90 95 100 105
F W A H Y A A A Q W D F G N T M C Q L L T
CCR5

ClaI*
BspDI* PvuI

gcctgtacttcattggcttcttctcaggcattttcttcatcatcctgctgaccatcgatcggtat 585
cggacatgaagtaaccgaagaagagtccgtaaaagaagtagtaggacgactggtagctagccata

110 115 120 125
G L Y F I G F F S G I F F I I L L T I D R Y
CCR5

SphI BglI BstEII PshAI

c t g g c t g t c g t g c a t g c c g t g t t c g c c c t g a a g g c a a g g a c c g t g a c a t t c g g a g t g g t g a c c t c 650

g a c c g a c a g c a c g t a c g g c a c a a g c g g g a c t t c c g t t c c t g g c a c t g t a a g c c t c a c c a c t g g a g

130 135 140 145

L A V V H A V F A L K A R T V T F G V V T S

CCR5

AleI

a g t c a t c a c a t g g g t g g t g g c c g t g t t t g c c t c c c t g c c c g g a a t c a t t t t c a c c c g g t c c c a g a 715

t c a g t a g t g t a c c c a c c a c c g g c a c a a a c g g a g g g a c g g g c c t t a g t a a a a g t g g g c c a g g g t c t

150 155 160 165 170

V I T W V V A V F A S L P G I I F T R S Q

CCR5

BpmI

a g g a g g g a c t g c a t t a t a c t t g c t c c a g c c a c t t c c c a t a c a g c c a g t a c c a g t t t t g g a a a a a c 780

t c c t c c c t g a c g t a a t a t g a a c g a g g t c g g t g a a g g g t a t g t c g g t c a t g g t c a a a a c c t t t t t g

175 180 185 190

K E G L H Y T C S S H F P Y S Q Y Q F W K N

CCR5

NmeAIII BsiHKAI SfcI

t t t c a g a c t c t g a a g a t c g t c a t t c t c g g c c t g g t g c t c c c a c t g c t g g t c a t g g t c a t t t g c t a 845

a a a g t c t g a g a c t t c t a g c a g t a a g a g c c g g a c c a c g a g g g t g a c g a c c a g t a c c a g t a a a c g a t

195 200 205 210

F Q T L K I V I L G L V L P L L V M V I C Y

CCR5

BsgI

t a g t g g t a t t c t g a a a a c c c t g c t g c g g t g c a g g a a c g a a a a g a a a c g g c a t a g g g c t g t g a g a c 910

a t c a c c a t a a g a c t t t t g g g a c g a c g c c a c g t c c t t g c t t t t c t t t g c c g t a t c c c g a c a c t c t g

215 220 225 230 235

S G I L K T L L R C R N E K K R H R A V R

CCR5

BspHI* BanI

t c a t c t t c a c t a t c a t g a t c g t g t a c t t t c t g t t c t g g g c a c c c t a c a a t a t c g t c c t g c t g c t c 975

a g t a g a a g t g a t a g t a c t a g c a c a t g a a a g a c a a g a c c c g t g g g a t g t t a t a g c a g g a c g a c g a g

240 245 250 255

L I F T I M I V Y F L F W A P Y N I V L L L

CCR5

PfoI* XcmI PaqCI

a a t a c c t t c c a g g a g t t c t t t g g c c t c a a c a a t t g t a g t a g t t c c a a c a g a c t c g a c c a g g c c a t 1040

t t a t g g a a g g t c c t c a a g a a a c c g g a g t t g t t a a c a t c a t c a a g g t t g t c t g a g c t g g t c c g g t a

260 265 270 275

N T F Q E F F G L N N C S S S N R L D Q A M

CCR5

MmeI BsaI PasI

g c a g g t g a c a g a g a c c c t g g g a a t g a c c c a c t g c t g c a t t a a t c c t a t t a t c t a c g c t t t c g t c g 1105

c g t c c a c t g t c t c t g g g a c c c t t a c t g g g t g a c g a c g t a a t t a g g a t a a t a g a t g c g a a a g c a g c

280 285 290 295 300

Q V T E T L G M T H C C I N P I I Y A F V

CCR5

ApoI BsrFI NgoMIV NaeI FseI

g c g a g a a a t t t c g g a a c t a c c t g c t g g t g t t c t t t c a g a a g c a c a t c g c a a a g a g g g g c c g g c c t 1170

c g c t c t t t a a a g c c t t g a t g g a c g a c c a c a a g a a a g t c t t c g t g t a g c g t t t c t c c c c g g c c g g a

305 310 315

G E K F R N Y L L V F F Q K H I A K R G R P

CCR5

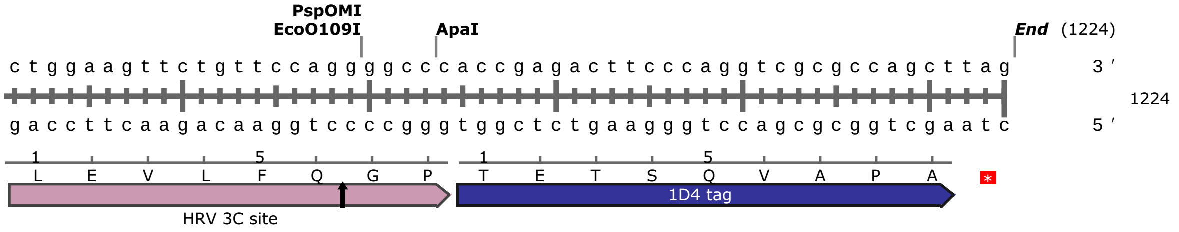

Supplementary Data 1: Annotated sequence of an example chemokine receptor (CCR5) construct (related to Figure 1)

The DNA sequence and the protein translation of the coding region for CCR5 insect cell expression pFastbac plasmid. The region includes the GP64 promoter, HA signal sequence, N-terminus 1X Flag tag, modified CCR5 receptor sequence with truncation from residue 320 at the C-terminus, 3C protease cleavage site and C-terminus 1D4 tag. This sequence is generated by SnapGene software.
